# Hepatoprotective and Anti-Inflammatory Activities of the* Cnidoscolus chayamansa* (Mc Vaugh) Leaf Extract in Chronic Models

**DOI:** 10.1155/2018/3896517

**Published:** 2018-07-25

**Authors:** Mariana Z. Pérez-González, A. Georgina Siordia-Reyes, Patricia Damián-Nava, Simón Hernández-Ortega, Martha L. Macías-Rubalcava, María A. Jiménez-Arellanes

**Affiliations:** ^1^Unidad, Investigación Médica en Farmacología, Hospital de Especialidades, CMN-SXXI, IMSS. Av. Cuauhtémoc 330, Col. Doctores, 06720 Ciudad de México (CDMX), Mexico; ^2^División Histopatología, Hospital de Pediatría, CMN-SXXI, Instituto Mexicano del Seguro Social (IMSS). Av. Cuauhtémoc 330, Col. Doctores, 06720 CDMX, Mexico; ^3^Instituto de Química, Universidad Nacional Autónoma de México (UNAM). Ciudad Universitaria, Delegación Coyoacán, 04510 CDMX, Mexico; ^4^Laboratorio de Rayos X, UNAM Ciudad Universitaria, Delegación Coyoacán, 04510 CDMX, Mexico; ^5^Departamento de Productos Naturales, UNAM Ciudad Universitaria, Delegación Coyoacán, 04510 CDMX, Mexico

## Abstract

Previous report described that CHCl_3_:MeOH extract of* C. chayamansa* leaves and pure compounds (moretenol, moretenyl acetate, kaempferol-3,7-dimethyl ether, and 5-hydroxy-7-3′,4′-trimethoxyflavanone) showed important topical and systemic anti-inflammatory activity in acute model, as well as* in vitro* antimycobacterial and antiprotozoal activities. In this paper, we describe the* in vivo* hepatoprotective and anti-inflammatory effects of the CHCl_3_:MeOH extract in chronic model and the isolation of additional compounds (moretenone and lupeol acetate). The hepatoprotective activity was determined at 39 days using Balb/c mice with liver damage induced with an antitubercular drug (RIF/INH/PZA). The anti-inflammatory activity was evaluated in a chronic model induced with CFA and in two acute models (TPA and carrageenan). In addition, moretenone and lupeol acetate were isolated and identified by spectroscopic data. Lupeol acetate is a main compound present in fractions 14-42, and this fraction was the majority fraction from the extract; from this moretenone was obtained. In animals with liver damage, the extract at 200 and 400 mg/kg induced better body weight gain with respect to positive control (Silymarin); in addition, the mice that received the extract at 200 mg/kg and Silymarin exhibited slight steatosis; however, the animals' livers at 400 mg/kg did not show steatosis. The extract and fractions 14-42 showed a good anti-inflammatory activity by TPA model (DE_50_ = 1.58 and 1.48 mg/ear) and by carrageenan model (DE_50_ = 351.53 and 50.11 mg/kg). In the chronic inflammatory test, the extract at three doses revealed a similar effect to that of phenylbutazone, although the body weight gain was better in animals that received the extract at 900 mg/kg.* Conclusion*. The CHCl_3_:MeOH extract showed significant anti-inflammatory and good hepatoprotective effect on chronic models. The fraction containing moretenone and lupeol acetate as main compounds was more active than extract in both acute models of inflammation.

## 1. Introduction

*Cnidoscolus chayamansa *(Mc Vaugh) belonging to Euphorbiaceae is known as “Mexican spinach”, and the widespread popular name utilized in Mexico is “chaya”. It has high nutritional value (contains vitamins, essential minerals, protein such as amino acids, and some fatty acids), more than* Spinacea oleracea* [1–3], and possesses important medicinal properties, including antioxidant, antitumoral, antimutagenic, antidiabetic, hypocholesterolemic, hepatoprotective, gastroprotective, and cardioprotective. From the CHCl_3_:MeOH extract of* C. chayamansa* leaves (collected in Hidalgo state, Mexico) two main terpenoids (moretenol and moretenyl acetate) and two polyphenols (kaempferol-3,7-dimethyl ether and 5-hydroxy-7-3′,4′-trimethoxyflavanone) were isolated. Terpenoids showed important topical and systemic anti-inflammatory activity, as well as* in vitro* antimycobacterial activity. The polyphenols demonstrated good antiprotozoal and anti-inflammatory activities [4]. In addition, this extract has LD_50_ >2 g/kg when administered intragastrically (i.g.) via Balb/C mice. The extract (1 g/kg) administered during 28 days by oral via on healthy Balb/C mice did not cause lethality, nor was body weight (BW) gain altered; biochemical parameters and histological analysis did not reveal any alteration. Other compounds isolated from this medicinal plant are quercetin, kaempferol, amentoflavone, nicotiflorin, astragalin, kaempferol-3-*O*-rutinoside, coumarin, naringenin, rutin, catechin, protocatechuic acid, and dihydromyricetin [3, 4].

With respect to hepatoprotective activity by* C. chayamansa*, there is only one report, to our knowledge, that supports this effect. The EtOH extract (at 200 and 400 mg/kg), administered i.g. via male Wistar rats with liver damage induced by antitubercular (antiTB) drugs [Rifampicin (RIF) and Isoniazid (INH) 100 mg/kg each one] was tested for 21 days, using as positive hepatoprotective control Silymarin (Sil, 2.5 mg/kg). This extract at 200 mg/kg decreases the serum concentrations of the hepatic enzymes, aspartate aminotransferase (AST), alanine aminotransferase (ALT), and alkaline phosphatase (ALP), as well as total protein and total albumin; these values were similar to Sil treated group. Histological analysis of rats' liver that received EtOH at 200 mg/kg demonstrated partial recovery in liver cellular structure, while the 400 mg/kg dose exhibited good hepatocyte integrity. The authors attributed this hepatoprotective effect to the antioxidant activity of the secondary metabolites contained in* C. chayamansa*, due to that antiTB drugs exert oxidative damage on liver tissue through free radical generation [5].

## 2. Materials and Methods

### 2.1. General Experimental Procedures

Chemical characterization of the isolated compounds was determined by Nuclear Magnetic Resonance (NMR) on Bruker-Avance III HD 700 equipment utilizing Tetramethylsilane (TMS) as an internal reference, in CDCl_3_ at 700 (^1^H) or 175 (^13^C) MHz. HRMS (DART-TOF+) was acquired with an AccuTOF-JMS-T100LC spectrometer (Jeol, Peabody, MA, USA). Single crystals of moretenone were obtained and their X-ray analysis was performed on a Bruker D8 Venture *κ* geometry diffractometer with a Cu-target microfocus X-ray source (*λ* = 1.54178 Å). A colorless crystal was mounted on a glass fiber at room temperature, the detector was placed at a distance of 5.0 cm from the crystals, and frames were collected with a scan width of 0.3 in w and an exposure time of 10 s/frame. Frames were integrated with the Bruker SAINT software package employing a narrow-frame integration algorithm. Monoclinic C system was used in systematic absences and intensity statistics in monoclinic C2 space-group determination for the moretenone compound.

The structure was solved utilizing direct methods using the SHELXS-2014/7 program. Anisotropic structure refinements were achieved employing the full matrix, least-squares technique on all nonhydrogen atoms. All hydrogen atoms were placed in idealized positions, based on hybridization, with isotropic thermal parameters fixed at 1.2 times the value of the attached atom. Structural refinements were performed using SHELXL-2014/7 [6]. Uncorrected melting points (m.p.) were determined in a Fisher-Johns apparatus. Gas Chromatography-Coupled Mass Spectra (GC-MS) analyses were performed on an Agilent Technology 6890N gas chromatograph interfaced with a Jeol MS-GCMATE II mass spectrometer. The GC column was a HP5 (30 m x 0.32 mm i.d.) programmed from 40 to 310°C at the rate of 8°C/min; the carrier gas was He (7 psi, 1 mL/min). Triterpenoids and sterols present in lipophilic fractions were identified by comparing their MS with those reported in the Publish/National Institute of Standards (NIST) MS Library.

Open Column chromatography on normal phase (CC-NP) was carried out on silica gel 60 (70-230 mesh; Merck, Darmstadt, Germany). Thin layer chromatography (TLC) analyses were performed on silica gel 60 F254 precoated aluminum plates (0.2 mm, Merck) and spots were visualized by spraying with a 10% aqueous H_2_SO_4_, followed by heating to identify triterpenoids and sterols; for flavonoid detection, methanolic diphenylboric acid-*β*-ethylamino ester and 5% ethanolic PolyEthylenGlycol-400 (NP/PEG) were employed [4].

### 2.2. Plant Collection and Extract Preparation

*Cnidoscolus chayamansa* (Mc Vaugh) was collected in the Miguel Hidalgo Delegation of Mexico City, Mexico, in June 2016. The plant was identified by M.Sc. Abigail Aguilar at the Herbarium, IMSS Mexico, and a voucher specimen (16252) was deposited at this herbarium. Dry leaves (316.4 g) were extracted successively by maceration at room temperature with CHCl_3_:MeOH 1:1 (CnCM). The extract was concentrated at 40°C in a vacuum system (BuchiVac V-153) and maintained at 25°C under conditions of darkness until its use.

### 2.3. Chemical Fractionation and Isolation of Pure Compounds

The CnCM extract (10 g) was subjected to CC-NP in silica gel 60 (200 g) and was eluted with Hex:EtOAc (100→0) and EtOAc:EtOH (100→0), following the procedure previously described [4]; from this procedure, a 24-fraction group was obtained. The least polar fractions, F1-12 and F14-42 (majority fraction from extract), were analyzed by GC-MS. The chromatogram of F1-12 showed the presence of three compounds: lupeol, octacosane, and stigmast-4-en-3-one (R*t* = 13.35, 14.57, and 20.55 min, respectively).

**Figure 1:**
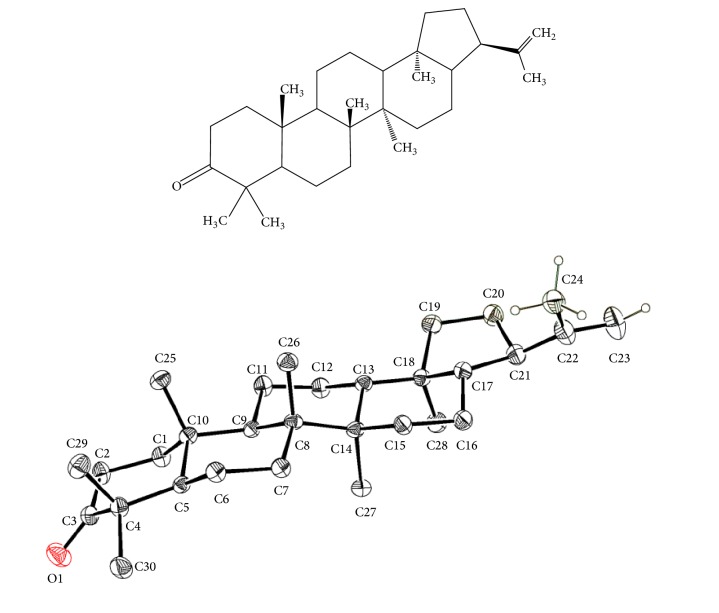
X-ray crystal structures of moretenone.

In primary fraction F14-42, main compounds were detected: *β*-amyrenone (R*t* = 16.81 min), *β*-amyrin acetate (R*t* = 17.25 min), moretenone (R*t* = 20.21 min), and lupeol acetate (R*t *= 20.95 min). In addition, three minor components were detected (ergost-5-en-3-ol, stigmasterol, and *β*-sitosterol with R*t* = 13.85, 14.50, and 16.45 min, respectively). Additionally, 2.8 g of primary fraction F14-42 was submitted to CC-NP in silica gel (54 g) eluted with Benzene 100% (F_1-17_), Hex 100% (F_18_), and Hex:CHCl_3_ 8:2 (F_21-23_). From secondary fraction F_10-23_ (eluted with Hex and Hex:CHCl_3_ 9:1)_,_ after two successive CC-NP, this was eluted with only hexane 100%; 250 mg transparent needle-shaped crystals with m.p. 220-225°C were obtained, which were soluble in CHCl_3_ and EtOAc, with R_*f*_ = 0.4 in Hex:EtOAc 10:0.26 when sprayed with H_2_SO_4_ 10%. This compound was identified as moretenone and all spectral data (MS, ^1^H-, and ^13^C-NMR) were in agreement with those described previously in the literature [7–9]. The main signal in ^1^H-NMR was (CD_3_OD): *δ* 2.42 (1H, m, H22), 4.72 (1H, s, H29 *α*), 4.70 (1H, s, H-29 *β*), 1.05 (3H, s, H23), 0.93 (3H, s, H24), 1.03 (6H, s, H25 y H26), 0.68 (3H, H28), and 1.52 (3H, m, H-30), and, in ^13^C-NMR, the main signal was *δ* 218.2 (C-3), *δ* 148.12 (C-22), *δ* 108.98 (C-29), and *δ* 19.78 (C-30). This compound was recrystallized in CH_2_Cl_2_ and the chemical structure was confirmed by X-rays (Figure 1). Crystal structure data was deposited at the Cambridge Crystallographic Data Center with deposit number CCDC 1574161 and molecular formula C_30_H_48_O_1_ (http://www.ccdc.cam.ac.uk).

### 2.4. Animal In Vivo Assays

Male Balb/C mice (22-25 g) were maintained under standard laboratory conditions according to Mexican Official Norm (NOM-062-ZOO-1999) modified in 2016. Animal received humane care according to the Guide for the Care and Use of Laboratory Animals prepared by National Academy of Science. The protocol was approved by the National Commission of Scientific Investigation, IMSS (CNIC R-2014-785-075).

### 2.5. Acute Topical and Systemic Anti-Inflammatory Activities

These tests were performed according to the previously described [4].

#### 2.5.1. Induced Mouse Ear Edema with 12-O-Tetradecanoyl Phorbol 13-Acetate (TPA)

TPA and CnCM extract were dissolved in acetone and applied topically. All test groups (n = 6) received 2.5 mg of TPA in the right ear (W's), while the left ear received only acetone (Wo); 30 min later, Indomethacin (IND) and CnCM extract or fractions were applied in the right ear at three doses (0.5, 1, and 2 mg/ear). Anti-inflammatory activity was calculated according to the weight difference between W's and Wo in ear sections (6 mm) at 6 h, employing the following formula [4]:

(1) %  Inhibition=W's−WocontrolW's−WosamplesW's−Wocontrol×100

#### 2.5.2. Carrageenan-Induced Mouse Paw Edema

Paw edema was induced by subcutaneous (s.c.) injection of 20 *μ*L of 2% carrageenan in saline solution. One hour prior to the injection of carrageenan, the treated groups (n = 7) received IND (10 mg/kg), CnCM extract (150, 300, and 600 mg/kg), or the fractions (50, 75 and 100 mg/kg) by i.g. route. Tested samples were solubilized in Tween 80:H_2_O (1:9) and the control group received only the vehicle. The percentage of inhibition was calculated by comparing the paw edema measurements at different times (1, 2, 3, 5, and 7 h) (Et) with the zero times values (Eo). Results were analyzed with a formula previously described [4]:

(2) %  Inhibition=Et−EocarrageenanEt−EotreatedEt−Eocarrageenan×100

### 2.6. Chronic Inflammatory Model Induced with Complete Freund's Adjuvant (CFA)

This assay was carried out according to that previously described (Gutiérrez-Rebolledo et al. [10, 11]), with modifications. All groups (n = 7) were injected subcutaneously with 25 *μ*L of CFA in right hind paw on days zero and 14 (reinjection). The groups received, by i.g. route, the following treatment: Phenylbutazone (PBZ, 100 mg/kg) or CnCM extract (200, 450, and 900 mg/kg) daily from day 7 to day 28. All samples were solubilized in Tween 80:H_2_O (1:9); the healthy and the arthritic groups received vehicle alone. Paw edema was measured at days 1, 7, 14, 21, and 28 (Et) utilizing a digital micrometer (Mitutoyo model 293-831), and the value of day zero (Eo) was determined. BW gain was also registered on the same days. Percentage of edema inhibition in each group was calculated from days 14 to 28 by comparison with the CFA group without treatment as follows:

(3) %  Inhibition=Et−EoCFA  groupEt−EoTreated  groupEt−EoCFA  group×100

### 2.7. Hepatoprotective Activity

This assay was determined using the methodology previously described [5, 12], with modifications. Four groups (n = 10) of randomly selected male Balb/C were used. The antiTB drugs are RIF/INH/Pyrazinamide (PZA) (50:50:100 mg/kg) and Sil (2.5 mg/kg) and were solubilized in Carboxymethyl Cellulose (CMC) at 5%, and the CnCM extract was solubilized in Tween 80:CMC 5% (5:95). The treatment was administered for 39 days by i.g. in a volume not exceeding 10 mL/kg.

The groups formed were the following: Group I: antiTB drugs plus vehicle; Group II: antiTB drugs plus Sil; Groups III and IV: antiTB drugs plus CnCM extract at 200 and 400 mg/kg, respectively. During the experimental period, BW gain was recorded from day zero and every 7 days throughout the experiment until day 39. On the final experimental day, the animals were fasted for 12 h prior to blood sampling without anesthesia by retroorbital puncture. Blood samples were collected to obtain serum; after that, the animals were sacrificed by dislocation and their liver was extracted for weighing and histological analysis. The parameters determined in serum were the following: urea, creatinine ALT, AST, ALP, cholesterol, triacylglyceride, and high-density lipoprotein (HDL). This analysis was carried out in Selectra II Analyser (Model Vitalab 2) automated equipment with commercial brand kits (RANDOX LAB).

### 2.8. Histological Analysis

These tests were performed according to previously described [4].

### 2.9. Oxidative Stress (OS) Parameters

The livers were obtained, and the latter were placed in an ice bath to determine OS biomarkers. Tissue samples (500 mg) were homogenized in phosphate-buffered saline solution (2 mL at pH 7.4), and one mL of each homogenate was centrifuged at 12,500* g* and 4°C/30 min; the activity of SOD and catalase (CAT) was determined from the supernatants by colorimetric methods [10, 13]. Lipid peroxidation (LPO) and protein carbonyl content (PCC) were evaluated from uncentrifuged homogenate samples. These tests were performed according to those previously described [14, 15].

### 2.10. Statistical Analysis

The SigmaPlot ver. 12.0 statistical software program (2011-2012) was utilized for analysis of results and graphic elaboration. Data are presented as standard error of the mean (SEM). BW gain and development of paw edema were analyzed with bifactorial analysis of variance (ANOVA) and with a post hoc Student-Newman-Keuls (SNK) test. Results of* p *<0.05 are considered statistically significant. For hematological analysis parameters and oxidative damage parameters in tissues, one-way ANOVA was employed with a post hoc SNK test in which* p *<0.05 was considered significant.

Development of paw edema in the carrageenan model was analyzed with bifactorial ANOVA and with a post hoc SNK test. Results of* p *<0.05 were considered statistically significant.

## 3. Results

### 3.1. Phytochemical Analysis

The chemical fractionation of the extract led to obtaining 24 groups of fractions with increasing polarity. Less polar fractions (F1-12 and F14-42) were submitted to GC-MS analyses. In the chromatogram from F1 to 12, lupeol, octacosane, and stigmast-4-en-3-one were detected as main constituents. In the chromatogram from F14 to 42, *β*-amyrenone (R*t* = 16.81 min), *β*-amyrin acetate (R*t* = 17.25 min), moretenone (R*t* = 20.21 min), and lupeol acetate (R*t=* 20.95 min) were detected as main components. This fraction was submitted to CC-NP and, after this procedure, we obtained moretenone (**1**). This compound was identified from its IR and ^1^H- ^13^C-NMR spectroscopic data. Single crystal diffraction confirmed the structure of the compound. Figure 1 presents an ORTEP drawing of this structure.

### 3.2. Anti-Inflammatory Activity by Systemic and Topical Model

Results in the carrageenan assay showed that the IND group exhibited 62.05% inhibition at 5 h, while CnCM at 150, 300, and 600 mg/kg showed an inhibition of 35.81, 44.05, and 59.26%, respectively, at the same time (Table 1). The anti-inflammatory effect was dose-dependent, showing an ED_50_ = 351.53 mg/kg. Primary fraction F12-42 revealed a better anti-inflammatory effect than the CnCM extract; in this case, the ED_50_ was lower (50.11 mg/kg versus 351.5 mg/kg). In this fraction by GC-MS analysis, *β*-amyrenone, *β*-amyrin acetate, moretenone, and lupeol acetate were detected as main components and the anti-inflammatory activity has been described for each compound.

The results of the topical anti-inflammatory effect of the CnCM extract showed an ED_50_ value of 1.58 mg/ear, while the IND group showed 2.14 mg/ear (Table 2); this extract was more active than the reference drug. Primary fractions 14-42 contain *β*-amyrenone, *β*-amyrin acetate, moretenone, and lupeol acetate and exhibited a significant anti-inflammatory effect; in this case, ED_50_ was 1.48 mg/ear.

### 3.3. Anti-Inflammatory Activity by Chronic Paw Edema Model Induced with CFA

**Figure 2:**
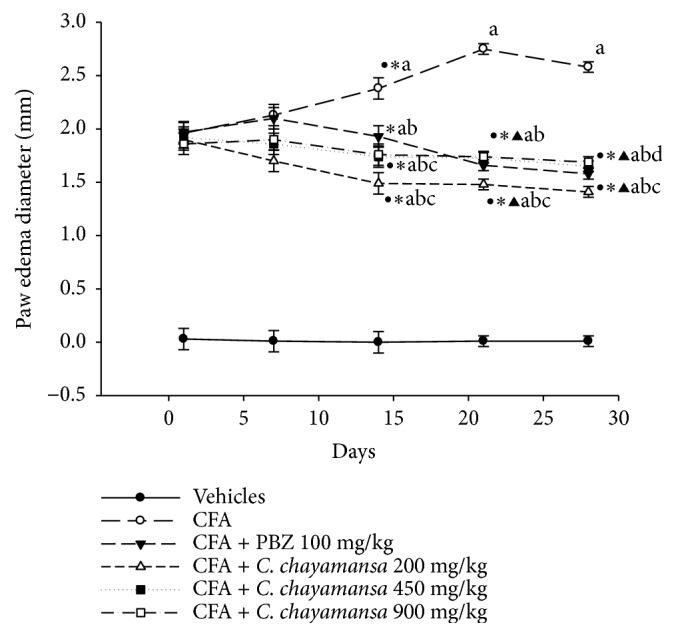
Effect of* C. chayamansa* extract on edema development (mm) during experimental chronic model in mice. Data shown as mean ± standard error (SEM). Treatments were administered daily by i.g. route from day 7 to 28. Two-way analysis of variance (ANOVA) of repeated measures (RM); post hoc Student-Newman-Keuls (SNK) (p≤0.05). ^a^vs vehicles; ^b^vs CFA control; ^c^vs CFA+PBZ; ^d^vs CFA+ CnCM 200 mg/kg; ^e^vs CFA+ CnCM 450 mg/kg; ^●^vs day 1; *∗*vs day 7; ^▲^vs day 14; ^■^vs day 21. CFA: Complete Freund's adjuvant; PBZ: phenylbutazone; n= 7.

The animals with chronic inflammation that received CnCM extract at 200 mg/kg exhibited a better anti-inflammatory effect from day 14 to day 28; at the end of the experiment, inhibition was 45.33% in this group, and the group that received PBZ (reference drug) showed 37.49%. Meanwhile, the group with the CnCM at 450 and 900 mg/kg revealed inhibition of ~35% at day 28; this effect was very similar, from day 14 to day 28, to that observed with PBZ. It is noteworthy that the anti-inflammatory effect observed for 200 mg/kg began from day 14 on and was maintained during the following 2 weeks; this effect was similar to that of PBZ (Figure 2).

**Figure 3:**
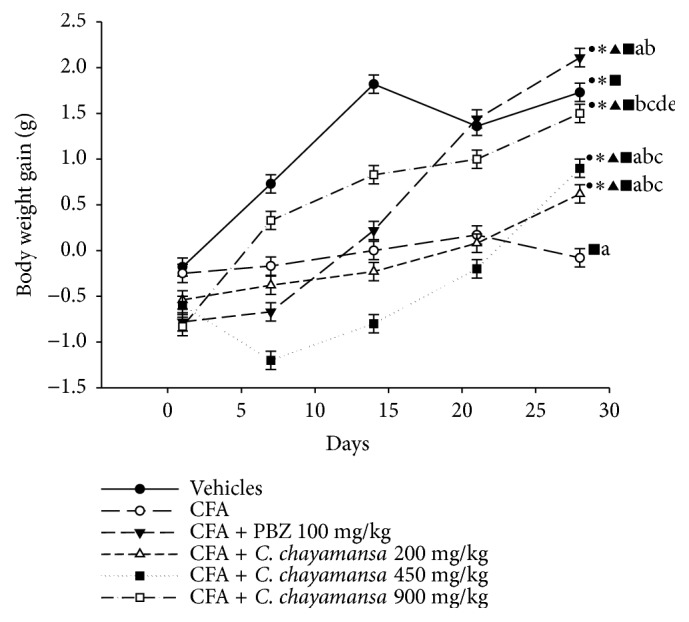
Effect of* C. chayamansa* extract on BW gain (g) during experimental chronic anti-inflammatory model in mice. Data shown as mean (±) standard error (SEM). Treatments were administered daily by intragastric route from day 7 to day 28. Two-way analysis of variance (ANOVA) of repeated measures (RM); post hoc Student-Newman-Keuls (SNK), (p≤0.05). ^a^vs vehicles; ^b^vs CFA control; ^c^vs CFA+PBZ; ^d^vs CFA+ CnCM 200 mg/kg; ^e^vs CFA+ CnCM 450 mg/kg; ^●^vs day 1; *∗*vs day 7; ^▲^vs day 14; ^■^vs day 21. CFA: Complete Freund's adjuvant; PBZ: phenylbutazone; CnCM: CHCl_3_:MeOH extract, n= 7.

In this model, BW gain also was registered by all groups (Figure 3). Animals with chronic inflammation showed poor BW gain with respect to healthy group (0.08 g versus 1.73 g); this loss in BW was more evident at day 28, and these animals exhibited lethargy and difficulty in walking. During the experiment period, animals with PBZ or CnCM extract at 900 mg/kg showed better BW gain (2.11 and 1.50 g, respectively); this behavior was similar to that of the healthy group (1.73 g), and this effect was more evident at day 28. This BW gain was more evident from day 21 in the PBZ group and from day 14 in the CnCM group at 900 mg/kg. On the other hand, the group treated with CnCM at 200 and 450 mg/kg exhibited similar values in BW gain (0.62 and 0.90 g) with respect to the CFA group at day 28 (0.8 g); BW gain was scarce in these groups with respect to the PBZ or vehicle groups (2.11 and 1.73 g, respectively).

### 3.4. In Vivo Hepatoprotective Effect

**Figure 4:**
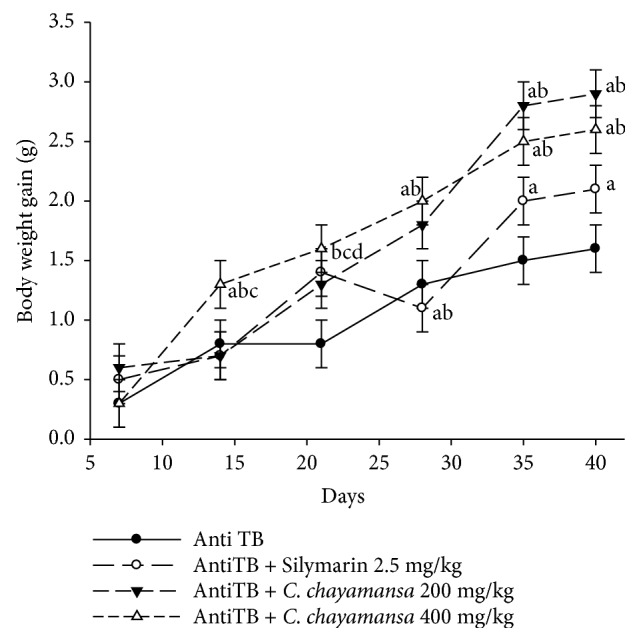
Effect of* C. chayamansa* extract on BW gain (g) during hepatotoxicity induced by antiTB drugs in Balb/C mice. Data presented as mean (±) standard error (SEM). Statistical analysis of variance (ANOVA); bifactorial of repeated measures (RM); post hoc Student-Newman-Keuls (SNK) test (*p*<0.05); ^a^vs AntiTB; ^b^vs AntiTB + Silymarin 2.5 mg/kg; ^c^vs AntiTB + CnCM 200 mg/kg; ^d^vs AntiTB + CnCM 400 mg/kg; AntiTB, antitubercular drugs (RIF/INH/PZA); n=7.

Mice with liver damage were treated for 39 days with the CnCM (200 and 400 mg/kg) or Sil. The results showed that the antiTB group and the Sil group had a scarce increase in BW gain at day 39; the values were 1.6 g and 2.1 g, respectively; this increase was slightly better in the Sil group. On the other hand, groups treated with CnCM extract demonstrated better BW gain; this increase was observed from day 14. BW gain was similar in both groups treated with the extract at 200 and 400 mg/kg (2.9 and 2.4 g, respectively), and this BW gain was better than that of the Sil and the antiTB groups (Figure 4).

**Figure 5:**
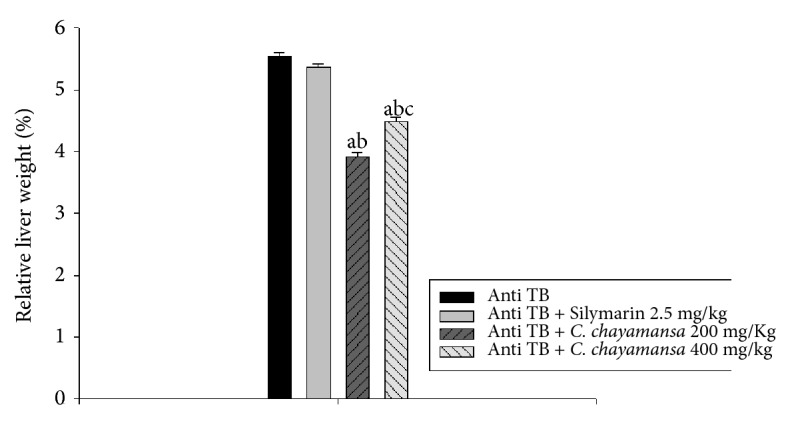
Effect of* C. chayamansa* extract over relative liver weight of Balb/C mice with hepatotoxicity induced by antiTB drugs. Data presented as mean (±) standard error (SEM). Statistical Analysis Kruskal-Wallis (ANOVA on Ranks) and post hoc Student-Newman-Keuls (SNK) test (p<0.05); ^a^vs AntiTB; ^b^vs AntiTB + Silymarin 2.5 mg/kg; ^c^vs AntiTB + CnCM 200 mg/kg; ^d^vs AntiTB + CnCM 400 mg/kg; AntiTB, antitubercular drugs (RIF, INH, PZA) mixture; CnCM: CHCl_3_:MeOH extract, n=7.

At day 39, all mice were euthanized and livers were extracted and weighed to calculate their relative weight compared to total animal weight. Results revealed that the antiTB and Sil groups exhibited a statistically similar value (1.55 g), while animals with CnCM at 200 mg/kg demonstrated a lower value (1.12 g) than that of the antiTB control (1.55 g). The CnCM extract group at 400 mg/kg also exhibited a value of 1.3 g; this value was lower than antiTB and Sil control (1.5 g for each group), but higher than the dose of 200 mg/kg (Figure 5).

The results of the biochemical analysis are described in Table 3, indicating that mice treated with Sil or the CnCM extract (at 200 or 400 mg/kg) showed an increase in the serum levels of the hepatic enzymes (AST, ALT, and ALP) with respect to the antiTB group, although an increase in hepatic enzymes values was observed; this cellular damage on the membranes could be confirmed in the histological analysis. It is noteworthy that, for renal function, both groups administered with the CnCM extract showed lower levels in the serum of creatinine and urea than the results exhibited by untreated mice with hepatotoxicity and in those animals treated with Sil. With respect to HDL values, the groups treated with the extract (both doses) and Sil showed lower values with respect to the antiTB group. Cholesterol values were similar in all treated groups and, for triacyl-glyceride values, mice treated with CnCM at both doses showed a significant decrease compared to the antiTB control.

Finally, the values of PCC and LPO are described in Table 4. The LPO levels in groups treated with both doses of the extract or with Sil were lower than in mice with liver damage caused with the antiTB drug. The levels of PCC for the CnCM extract (at both doses) were higher than those of the antiTB control and the Sil group.

For the antioxidant endogenous enzymatic results, only animals with hepatotoxicity and that were treated with* C. chayamansa* at 400 mg/kg showed a significant increase in the values of both tested enzymes: SOD with the highest activity of 54.56%, and CAT 91%, compared to antiTB mice (with liver damage); the antiTB plus Sil group showed a similar value to that of the antiTB group (Table 5).

**Figure 6:**
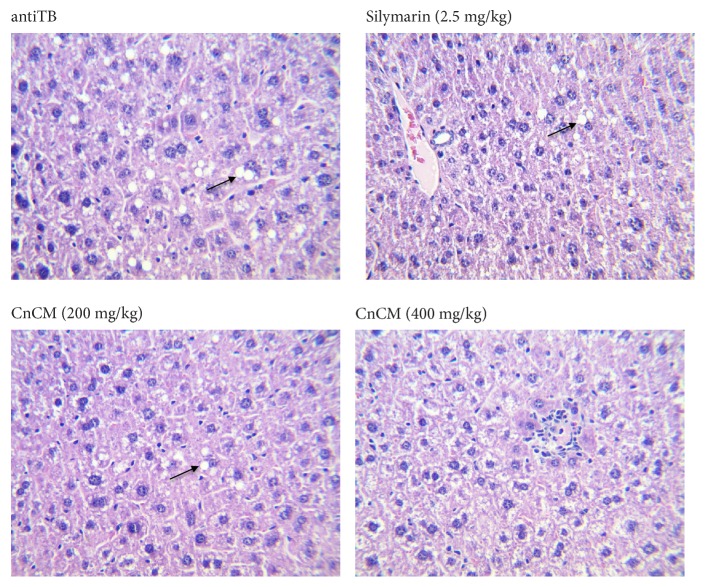
Histological liver section from Balb/C mice with hepatotoxicity induced by antiTB drugs and treated for 39 days with the CnCM extract. Histologic liver tissue (x100) collected from mice euthanized on day 39; after the treatment period, tissue were stained with hematoxylin and eosin 16X. AntiTB group showed steatosis, Silymarin (2.5 mg/kg) and CnCM (200 mg/kg) showed a moderate steatosis, and CnCM (400 mg/kg) group showed a scare steatosis.

On the other hand, in the histological analysis, the antiTB group exhibited moderate steatosis (3/3), without hepatic necrosis (0/3); the groups that received the extract at 200 mg/kg (2/3) and Sil (3/3) showed mild steatosis but without hepatic necrosis (0/3). It is important to mention that the group that received the extract at 400 mg/kg did not show steatosis (0/3), neither hepatic necrosis (0/3). On the other hand, only Sil group showed a slight portal lymphoid infiltrate (2/3). All groups did not show hepatic hematopoiesis; also they did not show centrilobular degeneration. These data are presented in Figure 6.

## 4. Discussion

In the CnCM extract, triterpenoids and sterols were detected as main compounds by TLC, and also scarce polyphenols were detected. Pérez-González et al. [4] previously described these types of compounds in the same extract from the plant material collected in the state of Hidalgo, Mexico. Chemical fractionation led to obtained moretenone from primary fractions F14-42 (majority fraction); this compound was obtained as a colorless crystal. Other main compounds detected in this fraction included *β*-amyrin acetate and lupeol acetate and *β*-amyrenone (a minor component).

For our knowledge, moretenone has not been reported for* C. chayamansa*, although its compound has been described as a semisynthetic derivate obtained from moretenol (isolated from* Cnidoscolus multilobus *aerial parts) by oxidative reaction [7]; this was isolated as a natural compound from the MeOH extract of* Maprounea guianensis* aerial parts [8], from* Euphorbia mellifera *MeOH extract [9], and from the MeOH extract of* Sebastiania schottiana* roots [16]. Previously, moretenol and moretenyl acetate were described from the same extract of* C. chayamansa* collected in the state of Hidalgo, Mexico [4]. Fractions 14-42 and the CnCM extract were subjected to topical and systemic anti-inflammatory assays; finding that this fraction was more active than the extract (ED_50_ = 50.11 and 351.53 mg/kg by carrageenan model, and ED_50_ = 1.48 and 1.58 mg/ear by TP assay, respectively).

Previously, moretenone was described as an analgesic and anti-inflammatory compound; the analgesic effect was higher than that of aspirin and paracetamol when determined by acetic acid induced abdominal writhes model (in male mice). The analgesic or antinociception activity (determined on acetic acid abdominal constrictions in mice) for moretenone was 72% inhibition; it was more active than aspirin and paracetamol (35 and 38% inhibition, respectively); on formalin assay, moretenone was more active (16 and 36% of inhibition at 1st and 2nd phases) than IND (6.6 and 33%, respectively). The analgesic activity determined on writhing assay and formalin assay, moretenone, showed ID_50_ = 8 and 42 *μ*mol/kg, respectively; it showed more activity than aspirin (133 and 123 *μ*mol/kg) or paracetamol (125 and 120 *μ*mol/kg, respectively) [16]. Also, anti-inflammatory activity by *β*-amyrin acetate and lupeol acetate has been described [15–18]; both compounds (*β*-amyrin acetate and lupeol acetate) exhibited 50% inhibitory dose (ID_50_) = 0.75 and 0.54 *μ*mol/ear on TPA model [17]. With respect to lupeol acetate, it showed a IC_50_ = 28.32 mg/kg in the antinociception effect (acetic acid induced abdominal writhes model); however, it was less active than IND (IC_50_ = 4.48 mg/kg) but was more active than aspirin (IC_50_ = 166.54 mg/kg). Another antinociception or analgesic model used was formalin-induced licking behavior; in this assay, lupeol acetate showed IC_50_ = 20.95 mg/kg. And* in vitro* anti-inflammatory activity (evaluated on LPS-stimulated RAW 264.7 cells line) was determined, the NO production, iNOS production and COX2 expression was inhibited at ID_50_ <5.13 *μ*M [18]. Another author described the* in vivo* anti-inflammatory effect by lupeol acetate; it has a significative anti-inflammatory activity by regulating TNF-*α* and IL-2 and involves the opioids system; in this study the author uses morphine as control drug; the assays were formalin test (2nd phase), carrageenan-induced paw edema, dextran-induced paw edema, and peritonitis induced by carrageenan [19].

In the case of this study, the anti-inflammatory result was better than that described previously for the same extract of* C. chayamansa *(ED_50_ = 467.73 mg/kg) collected in the state of Hidalgo [4]. Another study reported that only the EtOAc extract of* C. chayamansa* (collected in the Mexican state of Veracruz) administered by intraperitoneal route at 500 mg/kg (in CD1 mice) showed poor anti-inflammatory activity (30.29% of inhibition at hour 5) [2], while a similar anti-inflammatory effect was observed for the actual extract but at lower doses (at 150 mg/kg, 35.41%). Previously, a similar value (ED_50_ = 1.66 mg/ear) was described for the CnCM extract collected at a different site [4]. García-Rodríguez et al. [2] described the anti-inflammatory topical effect of the Hex, EtOAc, and EtOH; in this case, all extracts showed an inhibition of 31% at 2 mg/ear, an effect lower than that found in this work. Afterward, the CnCM extract was assayed on the chronic inflammatory model (induced with CFA); the animals with chronic inflammation were unable to walk or move because of the severe inflammatory process that they developed. The results showed that this extract at the three doses (200, 450, and 900 mg/kg) exerts a good anti-inflammatory effect. It is noteworthy that the anti-inflammatory effect on chronic inflammation observed for the 200 mg/kg dose starts from day 14 and was maintained during the following 2 weeks, and the decrease in the inflammatory process was similar to that exhibited by PBZ (Figure 2).

It is important to mention that there is, to our knowledge, no previous published work to date related to the activity of* C. chayamansa *on the chronic inflammation model. The results described previously demonstrated that inhibition over chronic edema development was not dose-dependent, due to the fact that the extract at a low dose (200 mg/kg) generates the best anti-inflammatory effect (45.33%) at day 28, even greater than PBZ (37.49%); however, this dose showed a low BW gain compared to that observed for the 900 mg/kg dose (0.62 g versus 1.5 g). Therefore, further studies will define which is the best recommended dose, and it is advisable to carry out studies at the molecular level to measure interleukins. It is important to mention that previously; two triterpenes and three polyphenols were isolated from* C. chayamansa* with an important anti-inflammatory effect on models of acute inflammation [4]. Perhaps these compounds are responsible for the effect observed in the model of chronic inflammation.

The present report is the first that describes anti-inflammatory activity of the CnCM on a chronic model; the results indicate that this extract, at the doses tested, shows a similar effect to that of the drug of reference (PBZ). Additional studies are being developed to determine the compounds responsible for this chronic anti-inflammatory activity.

With respect to the hepatoprotective effect, the most important finding was that the CnCM extract at 200 mg/kg generated better BW gain regarding animals with damage liver (antiTB group). On the other hand, animals were treated with the extract showed scare steatosis with respect to the Sil group and the antiTB group. Although the animals had hepatic damage caused by the antiTB drugs, the CnCM extract did not generate greater damage but, contrariwise, the animals presented less steatosis.

Previously, it was described that the EtOH extract from* C. chayamansa* possessed a hepatoprotective effect against liver damage induced with RIF/INH in rats [5]. In this study, it was found that the groups treated with extract or Sil reduced the levels of AST, ALT, and ALP with respect to those of the RIF/INH group. Previously it was described that the EtOH extract (*C. chayamansa*) at 200 exerted a slight hepatoprotective effect in rats with RIF/INH-induced liver damage and that this extract at 400 mg/kg demonstrated scarce necrosis, mild inflammation, and scarce steatosis. These authors concluded that the hepatoprotector effect exhibited by the extract was similar to that of the positive control (Sil). In addition, the hepatoprotective effect of the MeOH extract from* C. aconitifolius *on Wistar rats with liver damage caused by EtOH (chronic administration) has been described. In this case, the rats' liver slides treated with extract at 200 mg/kg revealed normal architecture without hepatic necrosis and without portal and central venous congestion. These alterations were more evident in rat damage with EtOH. The hepatoprotective effect observed for the MeOH extract was similar to control positive Kolaviron. This extract also showed an* in vivo* nephroprotective effect in the same model [20, 21].

## 5. Conclusions

*C. chayamansa* possesses good anti-inflammatory activity when used in the acute inflammation model (topical and systemic) and reveals a significant beneficial effect on the chronic inflammation model. In this case, the extract at 900 mg/kg favors BW gain; the effect was similar to that of PZB and also reduced the inflammation; this reduction was similar to that of the reference drug. In addition, the extract at both doses also showed good hepatoprotective effect against liver damage caused with antiTB drugs (RIF/INH/PZA). From this extract, moretenone and lupeol acetate from a less polar fraction were isolated as main compound, as well as a *β*-amyrin acetate.

## Figures and Tables

**Table 1:** Anti-inflammatory effect of *C. chayamansa* extract and primary fractions F14-42 on the development of carrageenan-induced acute inflammation.

Treatment 1	Dose (mg/kg)	T(5h)	Inhibition percentage	ED_50_ (mg/kg)

Carrageenan	-	0.95 ±0.03	-	-
IND	10	0.36 ±0.01^a^	62.05%	10

CnCM	150	0.61 ±0.01^ab^	35.81%	351.53 (R^2^ = 0.97)
	300	0.53 ±0.01^abc^	44.05%	
	600	0.39 ±0.01^acd^	59.26%	

Treatment 2				

IND	10	0.28 ±0.01^a^	63.22%	10

Primary fraction F14-42	50	0.39 ±0.02^a^	49.51%	50.11 (R^2^ = 0.93)
	75	0.19 ±0.01^ab^	74.47%	
	100	0.15 ±0.02^abc^	79.74%	

Data shown as mean (±), with standard error of the mean (SEM). Treatments were i.g. administered 1 h before the Carrageenan. Statistical analysis two-way ANOVA and post hoc SNK test (p ≤0.05). Treatment 1: ^a^vs Carrageenan control, ^b^vs Indomethacin, ^c^vs CnCM 150 mg/kg, and ^d^vs CnCM 300 mg/kg. Treatment 2: ^a^vs Carrageenan control. ^b^vs Indomethacin. ^c^vs fraction 14-42 75 mg/kg. ^d^vs fractions 14-42 100 mg/kg; CnCM: extract CHCl_3_:MeOH; IND: Indomethacin; n = 6.

**Table 2:** Anti-inflammatory effect of *C. chayamansa *extract and primary fractions F14-42 on the TPA model.

Treatment 1	Dose (mg/kg)	T(6h)	Inhibition percentage (%)	ED_50_ (mg/kg)

TPA	-	20.60±0.64	---	---
	0.5	16.92±0.38^a^	17.88%	
IND	1	14.07±0.40^•a^	41.77%	2.14
	2	10.77±0.34^•*∗*a^	47.73%	

CnCM	0.5	17.43±0.33^a^	15.37%	1.58
	1	11.48±0.57^•ab^	44.26%	
	2	9.45±0.98^•*∗*ab^	54.13%	

Treatment 2				

IND	0.5	8.62±0.23*∗*	45.64%	1.28
	1	7.9±0.44*∗*	50.18%	
	2	5.68±0.38*∗*^abd^	64.18%	

Primary fraction 14-42	0.5	9.42±0.56*∗*	15.37%	1.48 (R^2^=0.99)
	1	8.58±0.64*∗*	44.26%	
	2	7.46±0.46*∗*^b^	54.13%	

Data shown as mean (±), with standard error of the mean (SEM). Statistical analysis two-way ANOVA and post hoc SNK test (p ≤0.05). Treatment 1: ^a^vs TPA control, ^b^vs IND, ^c^vs CnCM 0.5 mg/ear, and ^d^vs CnCM 1 mg/ear. Treatment 2: ^*∗*^vs TPA control, ^a^vs IND 0.5 mg/ear, ^b^vs IND 1 mg/ear, ^c^vs fractions 14-42 0.5 mg/ear, and ^d^vs fractions 14-42 1 mg/ear; CnCM: extract CHCl_3_:MeOH; IND: Indomethacin; n = 6.

**Table 3:** Effect of *C. chayamansa* extract on the biochemical parameters of Balb/C mice with hepatotoxicity induced by antiTB drugs.

Parameters	Treatments			
	AntiTB	AntiTB + Silymarin	AntiTB + CnCM	

		2.5 mg/kg	200 mg/kg	400 mg/kg

Creatinine (mg/dL)	2.88±0.79	2.88±0.21	0.60±0.25^ab^	0.07±0.07^ab^
Urea (mg/dL)	51.07±5.63	68.45±5.00	50.63±8.43^b^	40.55±1.93^ab^
AST (IU/L)	198.00±17.45	256.33±25.32^a^	268.00±13.46^a^	317.00±8.62^abc^
ALT (IU/L)	175.00±32.25	228.33±31.09^a^	393.00±76.08^ab^	452.50±59.29^ab^
ALP (IU/L)	184.00±28.65	286.00±11.93^a^	252.67±38.27^a^	397.00±7.09^abc^
Cholesterol (mmol/L)	4.30±0.25	4.50±0.24	4.23±0.30	4.10±0.05
Triacyl-glyceride (mmol/L)	0.99±0.20	1.36±0.31	0.66±0.10	0.70±0.03
HDL (mmol/L)	1.30±0.06	1.05±0.07^a^	0.92±0.01^ab^	0.92±0.01^ab^

Data presented as mean (±) standard error of the mean (SEM). Statistical analysis one-way ANOVA, post hoc Student-Newman-Keuls (SNK) test (*p<*0.05); ^a^vs AntiTB; ^b^vs AntiTB + Silymarin 2.5 mg/kg; ^c^vs AntiTB + CnCM 200 mg/kg; ^d^vs AntiTB + CnCM 400 mg/kg; AntiTB: RIF/INH/PZA mixture; AST: aspartate aminotransferase; ALT: alanine aminotransferase; ALP: alkaline phosphatase; HDL: high density lipoprotein; n=7.

**Table 4:** Effect of *C. chayamansa* extract on oxidative stress biomarkers in Balb/C mouse liver tissue with hepatotoxicity induced by antiTB drugs.

Oxidative stress biomarkers	AntiTB	AntiTB + Silymarin 2.5 mg/kg	CnCM extract	
			200 mg/kg	400 mg/kg
PCC (*μ*m carbonyl/g)	403.81±31.28	397.46±26.44	610.16±14.30	517.94±28.87
LPO (*μ*m MDA/g)	177.82±2.21	126.67±5.64^a^	137.53±6.44^ab^	154.04±3.97^abc^

Data presented as mean (±) standard error of the mean (SEM). Statistical one-way ANOVA, post hoc Student-Newman-Keuls test (*p <*0.05); ^a^vs AntiTB; ^b^vs AntiTB + Silymarin 2.5 mg/kg; ^c^vs AntiTB + CnCM 200 mg/kg; ^d^vs AntiTB + CnCM 400 mg/kg; AntiTB: RIF/INH/PZA; PCC: protein carbonyl content; LPO: lipid peroxidation; MDA: malondialdehyde; n = 7.

**Table 5:** Effect of *C. chayamansa* extract on antioxidant enzymatic activity in Balb/C mouse liver tissue with hepatotoxicity induced by antiTB drugs.

Antioxidant enzymes	AntiTB	AntiTB+ Silymarin 2.5 mg/kg	CnCM extract	
			200 mg/kg	400 mg/kg
SOD (IU/g)	69.12±12.97	77.43±10.14	59.50±10.20	106.83±2.33^abc^
CAT (IU/g)	8.48±1.65	13.81±6.16	9.47±3.56	16.12±4.66^abc^

Data presented as mean (±) standard error of the mean (SEM). Statistical analysis, one-way ANOVA, and post hoc Student-Newman-Keuls (SNK) test (*p <*0.05); ^a^vs AntiTB; ^b^vs AntiTB + Silymarin 2.5 mg/kg; ^c^vs AntiTB + CnCM 200 mg/kg; ^d^vs AntiTB + CnCM 400 mg/kg; AntiTB: antitubercular drugs (RIF, INH, PZA); UI: international units; SOD: superoxide dismutase; CAT: catalase; n = 7.

## Data Availability

Crystallographic data for the moretenone has been deposited at the CCDC as supplementary publication CCDC 1574161. Copies of the data can be obtained, free of charge, from http://www.ccdc.cam.ac.uk/deposit@ccdc.cam.ac.uk. The data used to support the findings of this study are available from the corresponding author upon request.

## Abbreviations

ALT: Alanine aminotransferase
ALP: Alkaline Phosphatase
ANOVA: Analysis of Variance
antiTB: antitubercular
AST: Aspartate aminotransferase
BUN: Blood urea nitrogen
BW: Body weight
CAT: Catalasa
*C. chayamansa*: * Cnidoscolus chayamansa*
CCDC: Cambridge Crystallographic Data Center
CC-NP: Column chromatography on normal phase
CDCl_3_: Deuterated chloroform
CFA: Complete Freund's Adjuvant
CHCl_3_: Chloroform
CMC: Carboxymethyl Cellulose
CnCM: CHCl_3_:MeOH extract of* C. chayamansa*
ED_50_: Median effective dose
EtOH: Ethanol
EtOAc: Ethyl acetate
GC-MS: Gas Chromatography-coupled Mass Spectra
g: Gram
H_2_SO_4_: Sulfuric acid
Hex: Hexane
i.g.: Intragastric
IMSS: Instituto Mexicano del Seguro Social
INH: Isoniazid
kg: kilogram
LD_50_: Lethal dose medium
LPO: Lipid peroxidation
MeOH: Methanol
mL: Milliliter
min: Minute
M.Sc: Master Science
NIST: National Institute of Standards
NMR: Nuclear Magnetic Resonance
NP/PEG: Methanolic diphenylboric acid-*β*-ethylamino ester and 5% ethanolic PolyEthylenGlycol-400
OS: Oxidative stress
PCC: Protein carbonyl content
PBZ: Phenylbutazone
PZA: Pyrazinamide
RIF: Rifampicin
R*f*: Retention factor
R*t*: Retention time
SEM: Standard Error of the Mean
SNK: Student-Newman-Keuls
SOD: Superoxide dismutase
Sil: Silymarin
TLC: Thin layer chromatography
TMS: Tetramethylsilane (TMS).
